# Epidemiology and Trends of Sepsis in Young Adults Aged 20–44 Years: A Nationwide Population-Based Study

**DOI:** 10.3390/jcm9010077

**Published:** 2019-12-27

**Authors:** Carmen Bouza, Teresa López-Cuadrado

**Affiliations:** 1 Health Technology Assessment Agency, Carlos III Health Institute, 28029 Madrid, Spain; 2 National Centre of Epidemiology, Carlos III Health Institute, 28029 Madrid, Spain; teresalc@isciii.es

**Keywords:** sepsis, young adult, incidence, outcomes, trends

## Abstract

Background: While sepsis may have especially marked impacts in young adults, there is limited population-based information on its epidemiology and trends. Methods: Population-based longitudinal study on sepsis in adults aged 20–44 years using the 2006–2015 Spanish national hospital discharge database. Cases are identified by an ICD-9-CM coding strategy. Primary endpoints are incidence and in-hospital mortality. Trends are assessed for annual percentage change (AAPC) in rates using Joinpoint regression models. Results: 28,351 cases are identified, representing 3.06‰ of all-cause hospitalisations and a crude incidence of 16.4 cases/100,000 population aged 20–44. The mean age is 36 years, 58% of cases are men, and around 60% have associated comorbidities. Seen in one third of cases, the source of infection is respiratory. Single organ dysfunction is recorded in 45% of cases. In-hospital mortality is 24% and associated with age, comorbidity and extent of organ dysfunction. Incidence rates increase over time in women (AAPC: 3.8% (95% CI: 2.1, 5.5)), whereas case-fatality decline with an overall AAPC of −5.9% (95% CI −6.6, −5.2). Our results indicate that sepsis is common in young adults and associated with high in-hospital mortality, though it shows a decreasing trend. The substantial increase in incidence rates in women needs further research.

## 1. Introduction

Adult sepsis, defined as the dysregulated host response to infection resulting in organ dysfunction, is a major global health problem and represents a challenge for physicians and health care systems all over the world due to its high incidence, mortality and economic impact [1,2,3,4]. Additionally, survivors show a substantially worse quality of life, physical and cognitive decline and an increased risk of death several years after an episode [3,5].

The negative impact of sepsis, including a shortened lifespan and increased burden of morbidity and disability, can be especially significant in the case of young adults under 44 years of age as it is the population-group in which the majority of individuals are at their maximum physiological, biological and cognitive-intellectual capacity and supposes the active demographic, labor and economic force of the society.

There is limited population-based information on the epidemiology and trends of sepsis in these adults, however, especially in Europe where data from 2015 reveal that adults aged 20–44 years account for 34% of the population [6]. The size of this population and the results of several studies indicating that the incidence of sepsis has increased in the general population [4,7,8], make that information essential to assess, considering the burden of the disease and to estimate healthcare resource requirements. Thus, this population-based study was designed to address the epidemiological characteristics and trends of sepsis in young adults in Spain.

## 2. Materials and Methods

The data for this study were compiled from the Spanish Health Ministry’s National Minimum Basic Data Set (MBDS). Regarding the Spanish National Health System, when a patient is discharged from hospital, the responsible physician is required by law to record all diagnoses and clinical procedures performed according to the ICD-9-CM system. This information is then subjected to a validation process and consolidated in the MBDS. This database is considered to be representative of the national population as it includes data on over 90% of all hospitalizations produced annually in the country [9].

Viewing this database, each hospitalization is treated as a single registry and includes information on patient demographics, date of admission and whether elective or non-elective, date and destiny of discharge along with diagnostic codes including the main diagnosis, 13 additional diagnoses, and up to 20 procedures conducted during the patient’s hospital stay.

### 2.1. Case Identification

All hospitalizations of adults aged 20–44 years produced over a 10-year period from January 1, 2006 to December 31, 2015 were identified. To capture sepsis, we used an ICD-9-CM widely used strategy to define infection and organ dysfunction. To define infection, the following codes were used [7,8,10]: 038.0 (streptococcal septicaemia), 038.1 (staphylococcal septicaemia), 038.2 (pneumococcal septicaemia), 038.3 (septicaemia due to anaerobes), 038.4 (septicaemia due to other Gram-negative organisms), 038.8 (other specified septicaemias), 038.9 (unspecified septicaemia), 003.1 (salmonella septicaemia); 020.2 (septicaemia plague); 036.2 (meningococcal septicaemia); 036.3 (Waterhouse–Friderichsen syndrome); 054.5 (herpetic septicaemia); 098.89 (gonococcemia); 112.5 (systemic candidiasis); 112.81 (candidal endocarditis); 117.9 (other and unspecified mycoses) and 790.7 (bacteraemia). We also included the ICD-9-CM codes 995.91 (sepsis, systemic inflammatory response syndrome due to infectious process without organ dysfunction) and 995.92 (severe sepsis) [10].

To identify acute organ dysfunction, the ICD-9-CM codes detailed below were captured: Respiratory: 518.81 (acute respiratory failure), 518.82 (other pulmonary insufficiency), 518.84 (acute-on-chronic respiratory failure), 518.85 (acute respiratory distress syndrome after shock or trauma), 786.09 (respiratory distress, insufficiency), 799.1 (respiratory arrest), and 96.7 (invasive mechanical ventilation); Cardiovascular: 785.5 with all subcodes (shock without mention of trauma, includes 785.51, 785.52, 785.59); renal: 584 with all subcodes (acute renal failure), 580 (acute glomerulonephritis), and 39.95 (hemodialysis); Hepatic: 570 (acute and subacute necrosis of liver), 572.2 (hepatic coma), and 573.3 (hepatitis, unspecified); Haematologic: 286.6 (defibrination syndrome), 286.9 (other and unspecified coagulation defects), and 287.3–5 (secondary thrombocytopenia, unspecified); Neurologic: 293 (acute delirium), 348.1 (anoxic brain damage), 348.3 (encephalopathy, unspecified), 357.82 (critical illness polyneuropathy), 780.01 (coma), 780.09 (drowsiness, unconsciousness, stupor), and 89.14 (electroencephalogram); and Metabolic: 276.2 (acidosis metabolic or lactic).

Comorbidity was defined using the Charlson’s index adapted and validated by Deyo for the ICD-9-CM [11]. This index includes specific comorbid conditions of known prognostic value and has been shown effective to assess the mortality risk in patients with sepsis [12]. Further, due to the impact of comorbidity on the outcome of sepsis [13], cases were stratified according to the presence or not of comorbidities as defined by the Charlson’s index.

Concerning every case, the main diagnostic group at hospital admission was assessed according to the ICD-9-CM chapters: infectious disease (001–139); neoplasms (140–239); endocrine diseases (240–279); hematological diseases (280–289); neurological diseases (320–389); diseases of the circulatory system (390–459); respiratory diseases (460–519); diseases of the digestive system (520–579); diseases of the genitourinary tract (580–629); diseases of the skin and subcutaneous tissue (680–709); diseases of the musculoskeletal system and connective tissue (713–739) and injury-poisoning (800–999).

Episodes were described as surgical when this was indicated by their GRD (diagnosis-related group code). To identify specific microorganisms, code 041 was included as indicated by the ICD-9-CM coding manual for the purpose of identifying specific microorganisms in the case of diseases classified under the heading ‘other’ [9]. Hospitalization costs were estimated from their respective GRDs.

As the data examined were sourced from anonymized records, there was no need for informed consent [14].

### 2.2. Data Analysis and Presentation

During this descriptive study, the main variables examined were the incidence and mortality of sepsis and their trends over the study period. Other variables examined were demographics, comorbidities, microbiological data, number and type of organ dysfunction, and the use of hospital resources (length of hospital stay and costs).

Categorical variables are provided as their absolute frequencies and percentages, and continuous variables as means and standard deviations. Age groups were defined as 5-year intervals. In-hospital case-fatality rate (CFR) was calculated as the number of deaths divided by the number of cases of sepsis and expressed as a percentage. To identify factors associated with in-hospital mortality, an exploratory logistic regression was performed including variables of clinical relevance such as sex, age group and comorbidity burden, and others of known impact on the outcome of sepsis such as number of organ dysfunctions. Results are presented as odds ratios (OR) and 95% confidence intervals (CI).

Crude incidence rates were calculated using national population data, of those aged 20–44 years, from the Spanish institute of statistics (INE) and results expressed per 100,000 population [15]. Age-adjusted rates were calculated by direct standardization referred to the European population [16].

To assess possible temporal changes in incidence and in-hospital CFR, we used Joinpoint regression models [17]. These generalized linear models follow a Poisson distribution and served to estimate annual average percentage change (AAPC) and its 95% CI for each trend [18].

All statistical tests were performed using the package Stata^®^ version 15 (StataCorp LP, College Station, Texas, USA) and Joinpoint regression programme version 4.7.0.0 (National Cancer Institute, Bethesda, MD, USA). Significance was set at *p* < 0.05.

### 2.3. Data Availability

The data came from anonymized registries. According to the confidentiality agreement signed with the Ministry of Health, Consumer Affairs and Social Welfare, the data from this study cannot be shared with third parties. The authors did not have special access privileges. Should any researcher wish to gain access to these data, they can do so by applying directly to the Ministry through the following link: https://www.mscbs.gob.es/estadEstudios/estadisticas/estadisticas/estMinisterio/SolicitudCMBDdocs/Formulario_Peticion_Datos_CMBD.pdf.

## 3. Results

Regarding 9,271,272 hospital discharge registries corresponding to adults aged between 20 and 44 years for the period 2006–2015 in Spain, 28,351 episodes of sepsis were identified, representing 3.06‰ of all-cause hospitalizations in this age group.

**Figure 1:**
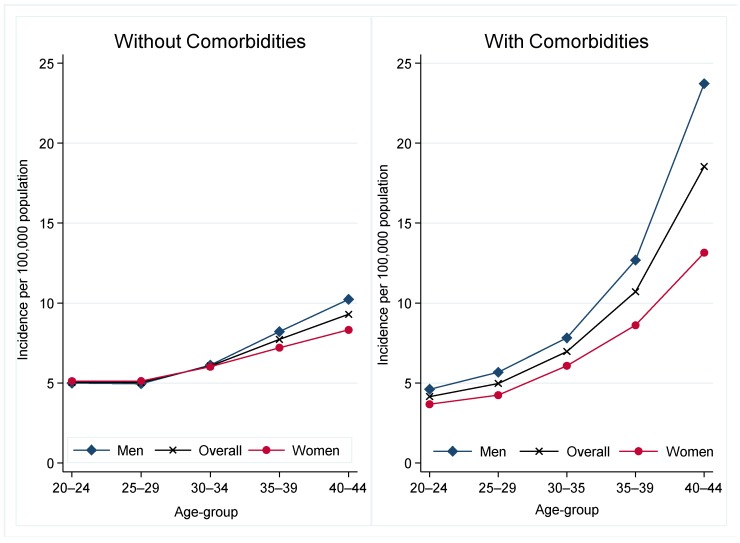
Incidence of sepsis according to the presence or absence of comorbidities, and age.

The crude incidence rate for the cohort was 16.36 cases per 100,000 persons; being higher in men (18.62 per 100,000 persons) than women (13.9 per 100,000 persons). Shown in Figure 1, incidence increased with age in cases with and without comorbidities, but the increase was greater among those with comorbidities. The increase with age was observed both in men and women, although in almost every age group the incidence was higher among men.

The overall mean age was 36 years and 58% of cases were men. Roughly 41% of cases (38.2% in men, 46% in women) had a Charlson score of 0. The most frequent comorbidities in the remaining cases were liver disease, cancer and AIDS (Table 1). This table also shows the demographic and clinical characteristics of the episodes stratified according to the presence or absence of comorbidities. Regarding most cases, hospital admission was non-elective via the emergency services and, in one third of cases, the cause of hospitalization was infection.

The potential source of infection in most cases was respiratory, followed by genitourinary and procedure-related infections. Found in close to 60% of cases, at least one microorganism was identified. Gram-negative bacteria were slightly more frequent than other microorganisms. The presence of bacteraemia was recorded in 21% (*n* = 5908) of the episodes, this rate being higher in those with comorbidities (24% versus 16.5%).

Seen in around 45% of cases, single organ dysfunction was present, while 26% and 25% showed the dysfunction of two or more organs, respectively. Shown in Table 1, the percentage of cases with more than two organ dysfunctions was greater among those with comorbidities. The most frequently affected organs were the lungs, recorded in more than half of the cases, followed by the cardiovascular system and kidneys. Renal dysfunction was much more frequent in the subset with comorbidities. Essentially, of the 3191 cases in which patients underwent dialysis, 2444 (77%) belonged to this subset. The use of invasive mechanical ventilation was, nevertheless, more frequent in the group without comorbidities and, overall, this measure was employed in 38% of cases.

The mean hospital stay was 28.4 days and the mean cost per case was 17,878 Euros. There were no significant differences between the cases with or without comorbidities.

Six thousand, eight hundred and eight hospital deaths were recorded, which corresponds to an overall case-fatality rate CFR of 24%, but this rate varied according to various demographic and clinical characteristics. Thus, as shown in Table 2, it was higher in men than in women and clearly increased with age.

The multivariate logistic regression analysis revealed that a greater age, the failure to detect the source of infection, the non-identification of the responsible microorganism, the extent of organ dysfunction and the presence of comorbidities were significantly associated with an increased risk of mortality (Table 2). When we perform the same analysis stratified by the presence or absence of comorbidities, as indicated by Charlson’s index, it is observed that, except for sex, mortality risk factors are the same with small differences in the odds ratios ORs (Table 3).

### 3.1. Trends

#### 3.1.1. Incidence

Regarding the whole population analyzed, and as shown in Table 4, the incidence of sepsis has discretely increased over the six years of the study, though not significantly. When adjusted by sex, there was no significant change in men, however, a significant increase was detected in the incidence rate in women.

Rates also increased significantly in cases with a Charlson’s index of zero but not in those with comorbidities. Additionally, our data show a decreasing rate over time of cases with one or two organ dysfunctions.

#### 3.1.2. In-Hospital Mortality

The temporal analysis indicated a significant drop in case-fatality rate CFR both overall and in each of the subsets examined (Table 4), however, the decrease produced was variable and lower in men than women. Similarly, Table 4 reveals that the fall was significant both in cases with a Charlson’s index of zero, as in those with comorbidities, although it was greater in the former. When we assessed the changes produced in CFR by number of organ dysfunctions, this variable was observed to fall in all cases, although the decrease was lower in the case of the dysfunction of more than two organs.

## 4. Discussion 

This is the first study to provide representative national estimates of epidemiological characteristics and incidence and mortality trends of sepsis in young Spanish adults, to our knowledge. Our findings reveal that sepsis is a common and frequently fatal condition among adults aged 20–44 years. Trends indicate that incidence rates are increasing in women but not in men, whereas in-hospital mortality is decreasing. Additionally, sepsis associates with a substantial use of hospital resources.

The incidence of sepsis observed was 16.4 cases per 100,000 persons aged 20–44 years. Our data confirmed that, even in this young age group, its frequency was defined by patient age [19] and was very much higher in individuals aged 40–44 years than in those aged between 20 and 24 years. Sepsis also more frequently affects men than women [4,7], something that literature has linked to a distinct immune response between men and women, suggesting an advantageous response from women to an infection [19]. However, in contrast with the findings of others who report an overall increase in incidence rates of sepsis in the general population [4,7,8], in our study only women showed a significant increase over the 10-year study period. Regrettably, the characteristics of our dataset do not permit us to identify the causes of this particular increase in women, nor if it may be related to differences in individual risk patterns or care-level determinants, but we feel that this finding merits further inquiry.

Among the most relevant findings of our study was the high in-hospital mortality associated with sepsis in the young adult, which amounted to 24% of all episodes. Mortality was higher in men than women and also shows a clear association with age and, especially, with the presence of comorbidities and the number of organ dysfunctions. Accordingly, age acts as an independent risk factor of mortality [20] even in the young adult and, as our data show, its impact is aggravated by the presence of comorbidities.

Though the capacity of comorbidities to affect the risk of sepsis remains unclear [21], our observation that around 60% of the cases in our cohort showed comorbidities with the predominance of liver disease, cancer and AIDS, confirms the results of others who suggest that chronic diseases, specifically those mentioned here, increase this risk [13,20,21]. Besides a high proportion of men and a difference in age, which was greater among cases with comorbidities, the most appreciable differences between those with and without comorbidities was a greater extent of organ dysfunction along with a greater frequency of renal dysfunction and a higher mortality in the former. This corroborates the fact that the presence of comorbidities promotes the development of multiple organ dysfunction [22] and has a great influence on the outcome of patients with sepsis [13,23]. Found in our study, this factor increased the risk of death by 2.82-fold.

We should highlight, however, the elevated in-hospital mortality observed for cases without comorbidities. This was 13.5% and much higher than that associated with other diseases in the 20–44 years age group in our country [9]. Further, we must also highlight that the proportion of cases without comorbidities has increased over time. Although we cannot establish a formal cause for this rise, it may be related to the increasing incidence in women who constitute a group with less comorbidity burden as measured by the Charlson Index.

Additionally, it was especially noticeable that both in cases with or without comorbidities, the presence of two or more organ dysfunctions was a critical event with a cumulative impact on the risk of death in the septic young adult [24].

The mortality observed here doubles the 12% described by Kaukonen [25], who analyzed the data of 15,471 cases of severe sepsis in patients aged ≤44 years in a retrospective observational study performed in the intensive care units (ICUs) of Australia and New Zealand between 2000 and 2012. This discrepancy could be explained in part by differences in information systems, methodology and setting. Although Kaukonen analyzed cases from ICUs and we looked at a national-level population, notable clinico-demographic differences were observed with a profile that suggests greater severity and mortality risk in our study, as our cases were older and showed greater pulmonary, cardiovascular and renal organ dysfunction and a greater comorbidity burden. Regarding this last issue, in the absence of comorbidities, the mortality observed in the present study is greater than the 8% described by Kaukonen, although the systems used to assess comorbidities differed between the two studies.

Both studies coincided, however, in observing a significant decline in hospital mortality rates of sepsis in patients aged ≤44 years. Considering our study, we observed from 2006 to 2015 an annual reduction in hospital mortality of 5.9%, a similar figure to that noted by Kaukonen. This decrease in mortality was observed both in men and women but was less marked in cases with comorbidities and in those showing two or more organ dysfunctions.

Concerning resource utilization, both mean hospital stay and costs were elevated and much above the mean figures provided by the National Health Service for all-cause hospitalizations in this age group. Over the period analysed, these figures were 4.9 days of mean stay and 3219.26 Euros per hospitalization [9]. We could easily speculate that this can be related to the use of costly invasive therapeutic interventions in sepsis [26,27].

While sepsis is often associated with elderly subjects, this study indicates that it is frequent and related to a high in-hospital mortality in young adults. According to data from the UN, adults aged 20–44 years in 2015 accounted for 33.7% of the Spanish population [8]. This means sepsis in this age group is a real challenge for clinicians and healthcare systems. Our findings identify a need to implement measures designed to optimize its prevention and management, including early diagnostic and therapeutic measures, especially in high-risk patients with comorbidities, targeted at reducing the development and progression of organ dysfunction and, consequently, mortality. Essentially, as comorbidities are usually easily recognizable chronic conditions, if these are managed early on and adequately, it could be possible to improve outcomes in these patients [28].

Clinical-administrative databases are an essential tool for health research [16,29]. Spain has a large population database which is required by law and is widely representative as it covers practically all hospitalizations produced annually, allowing for accurate epidemiological estimates. During this study, we followed RECORD guidelines for observational studies’ routinely-collected health data [30]. Notwithstanding, due to its inherent characteristics, the database employed has some limitations. Although national guidelines exist for the use of the coding system, this may not be uniform across the national healthcare system and we cannot rule out coding errors, despite regular audits; however, we consider systematic coding errors highly unlikely. Moreover, due to its confidential nature, the database used lacks complete clinical information, precluding any causal inferences. The use of national databases is, nevertheless, well established for the epidemiological monitoring of the incidence and mortality of sepsis [31,32]. Additionally, the results of a recent meta-analysis confirm the essential role of administrative data for surveillance of mortality trends in sepsis [33]. Another limitation is that studies based on hospital discharge data do not include non-hospitalized cases of sepsis such that our estimates of incidence and mortality are conservative. Similarly, deaths produced after hospital discharge also will be missed [5,34].

## 5. Conclusions

This population-based nationwide study shows that sepsis is a common, and frequently fatal, condition among adults aged 20–44 years. Data over time indicate that incidence rates are increasing in women but not in men and in cases without comorbidity, findings that need further research. Conversely, in-hospital mortality shows a decreasing trend. This trend was lower in cases with comorbidity and dysfunction of more than two organs. Additionally, sepsis associates with a substantial use of hospital resources.

## Figures and Tables

**Table 1:** Characteristics and outcomes of sepsis.

	All Cases (*n* = 28,351)	Without Comorbidities * (*n* = 11,798)	With Comorbidities * (*n* = 16,553)	*p*-Value
Age, mean ± SD	35.73 ± 6.63	34.59 ± 6.83	36.55 ± 6.37	<0.001
Age group, years				<0.001
20–24	2381 (8.4)	1307 (11.1)	1074 (6.5)	
25–29	3217 (11.4)	1619 (13.7)	1598 (9.7)	
30–34	4971 (17.5)	2313 (19.6)	2658 (16.1)	
35–39	7287 (25.7)	3054 (25.9)	4233 (25.6)	
40–44	10,495 (37.0)	3505 (29.7)	6990 (42.4)	
Sex				<0.001
Men	16,501 (58.2)	6299 (53.4)	10,202 (61.6)	
Women	11,848 (41.8)	5498 (46.6)	6350 (38.4)	
Non-Elective Hospital Admission	24,988 (88.1)	10,916 (92.5)	14,072 (85.0)	<0.001
Medical Admission	18,543 (65.5)	11,312 (68.4)	7231 (61.4)	<0.001
Hospital Admission Main Diagnosis (ICD-9-CM chapter)				
Infectious disease	9164 (32.3)	3844 (32.6)	5320 (32.1)	0.432
Respiratory disease	3774 (13.3)	1874 (15.9)	1900 (11.5)	<0.001
Trauma & poisoning	3640 (12.8)	2066 (17.5)	1574 (9.5)	<0.001
Digestive disease	2938 (10.4)	1241 (10.5)	1697 (10.2)	0.467
Cancer	2137 (7.5)	94 (0.80)	2043 (12.3)	<0.001
Genitourinary disease	1560 (5.5)	874 (7.4)	686 (4.1)	<0.001
Circulatory disease	1372 (4.8)	186 (1.6)	1186 (7.2)	<0.001
Charlson Score, mean ± SD	1.77 ± 2.29	NA	3.03 ± 2.27	
Specific Comorbidities ^‡^				
Liver disease	5670 (20.0)		5670 (34.2)	
Cancer	4320 (15.2)		4320 (26.1)	
AIDs	2298 (8.1)		2298 (13.9)	
Chronic kidney disease	2327 (8.2)		2327 (14.1)	
COPD	1714 (6.1)		1714 (10.4)	
Cardiac insufficiency	1588 (5.6)		1588 (9.6)	
Diabetes	1646 (5.8)		1646 (9.9)	
Hemiplegia or Paraplegia	1213 (4.3)		1213 (7.3)	
Stroke	1052 (3.7)		1052 (6.4)	
Rheumatologic disease	475 (1.7)		475 (2.9)	
Peripheral vascular event	373 (1.3)		373 (2.2)	
Acute myocardial infarction	234 (0.8)		234 (1.4)	
Infection Site ^‡^				
Respiratory	8414 (29.7)	3808 (32.3)	4606 (27.8)	<0.001
Genitourinary	4953 (17.5)	2439 (20.7)	2514 (15.2)	<0.001
Procedure-related	4352 (15.4)	1830 (15.5)	2522 (15.2)	0.526
Abdominal	3271 (11.5)	1512 (12.8)	1759 (10.6)	<0.001
Soft tissue	1265 (4.5)	549 (4.7)	716 (4.3)	0.188
Central nervous system	799 (2.8)	369 (3.1)	430 (2.6)	0.008
Cardiac	375 (1.3)	124 (1.1)	251 (1.5)	0.001
Other/Not specified	7112 (25.1)	2625 (22.3)	4487 (27.1)	<0.001
Microbiological Data ^‡^	16,225 (57.2)	6568 (55.7)	9657 (58.3)	<0.001
Gram-positive bacteria	8340 (51.4)	3336 (50.8)	5004 (51.8)	
Gram-negative bacteria	9457 (58.3)	3959 (60.3)	5498 (56.9)	
Fungus	815 (5.0)	266 (4.1)	549 (5.7)	
No. Organ Dysfunctions ^†^				<0.001
1	12,764 (45.0)	5367 (45.5)	7397 (44.7)	
2	7365 (26.0)	3274 (27.8)	4091 (24.7)	
>2	7095 (25.0)	2565 (21.7)	4530 (27.4)	
Type of Dysfunction ^‡^				
Respiratory	15,159 (53.4)	6560 (55.6)	8599 (51.9)	<0.001
Cardiovascular	13,178 (46.5)	5671 (48.1)	7507 (45.4)	<0.001
Renal	10,800 (38.1)	3713 (31.5)	7087 (42.8)	<0.001
Haematological	5652 (20.0)	2383 (20.2)	3269 (19.8)	0.350
Neurological	3145 (11.1)	1358 (11.5)	1787 (10.8)	0.059
Hepatic	2491 (8.8)	none	2491 (15.1)	<0.001
Metabolic	2471 (8.7)	1025 (8.7)	1446 (8.7)	0.888
Invasive Therapeutic Measures				
Mechanical ventilation	10,056 (35.5)	4481 (38.0)	5575 (33.7)	<0.001
Haemodyalisis	3191 (11.3)	747 (6.3)	2444 (14.8)	<0.001
In-Hospital Death	6808 (24.0)	1588 (13.5)	5220 (31.5)	<0.001
Hospital LOS, d, mean ± SD	28.44 ± 37.70	28.06 ± 37.90	28.72 ± 37.55	0.149
Hospitalization Cost, Euros mean ± SD	17,878 ± 22,348	17,888 ± 23,486	17,871 ± 21,501	0.949

* Comorbidity is defined by the Charlson Index. Cases with a Charlson Index of 0 are considered ”without comorbidities”. Cases with a Charlson index >0 are considered “with comorbidities”. Comparisons are made between cases without and with comorbidities (*p*-Value). Values in parentheses are percentages. SD: standard deviation. COPD: chronic obstructive pulmonary disease. LOS: length of hospital stay. ^‡^ Subgroups not mutually exclusive. d: days. ^†^ Not specified No. organ dysfunctions: 1127 cases (3.98%).

**Table 2:** In-hospital deaths. General characteristics, case-fatality and risk (*n* = 6808).

Characteristic	Cases	Case-Fatality Rate (% Severe Sepsis)	Bivariate OR (95% CI)	Multivariate OR (95% CI), *p*-Value
Sex				
Women	2455	20.72	Reference group	Reference group
Men	4353	26.38	1.37 (1.30, 1.45)	1.12 (1.05, 1.19)
Age-Group (years)				
20–24	405	17.01	Reference group	Reference group
25–29	611	18.99	1.14 (0.99, 1.31)	1.10 (0.94, 1.28), 0.224
30–34	1050	21.12	1.31 (1.15, 1.48)	1.19 (1.03, 1.36), 0.016
35–39	1738	23.85	1.53 (1.36, 1.72)	1.28 (1.13, 1.47), <0.001
40–44	3004	28.62	1.96 (1.74, 2.19)	1.48 (1.30, 1.68), <0.001
Charlson Index Comorbidity				
0	1588	13.46	Reference group	Reference group
>0	5220	31.54	2.96 (2.78, 3.15)	2.82 (2.63, 3.01), <0.001
Diagnostic Categories				
Medical	4457	24.04	Reference group	Reference group
Surgical	2338	23.91	0.99 (0.94, 1.05)	Not applicable
Pathogens Identified				
No	3663	30.21	Reference group	Reference group
Yes	3145	19.38	0.56 (0.53, 0.59)	0.68 (0.64, 0.72), <0.001
Principal Site of Infection				
Respiratory	2179	25.90	1.16 (1.09, 1.23)	1.15 (1.06, 1.25), 0.001
Abdominal	804	24.58	1.04 (0.95, 1.13)	Not applicable
Genitourinary	555	11.21	0.35 (0.32, 0.38)	0.53 (0.48, 0.60), <0.001
Soft tissue	223	17.63	0.67 (0.58, 0.77)	0.80 (0.67, 0.94), 0.007
Procedure-related	755	17.35	0.62 (0.57, 0.68)	0.80 (0.73, 0.89), <0.001
Not specified	2483	34.91	2.10 (1.98, 2.23)	1.69 (1.55, 1.84), <0.001
No. Organ Dysfunctions				
1	1634	12.80	Reference group	Reference group
2	1850	25.12	2.28 (2.12, 2.46)	2.27 (2.10, 2.45), <0.001
>2	3171	44.69	5.50 (5.13, 5.90)	5.06 (4.70, 5.45), <0.001

OR, odds ratio; 95% CI, 95% confidence interval.

**Table 3:** In-hospital mortality according to the presence or absence of comorbidities.

Characteristic	Multivariate OR (95%CI), *p*-Value	
	Without Comorbidities	With Comorbidities
Sex		
Women	Reference group	Reference group
Men	1.27 (1.13, 1.43), <0.001	1.06 (0.98, 1.14), 0.146
Age-group (yrs)		
20–24	Reference group	Reference group
25–29	1.09 (0.85, 1.39), 0.508	1.11 (0.92, 1.35), 0.274
30–34	1.20 (0.95, 1.51), 0.118	1.19 (0.99, 1.41), 0.059
35–39	1.14 (0.92, 1.42), 0.228	1.35 (1.14, 1.60), <0.001
40–44	1.44 (1.16, 1.77), 0.001	1.51 (1.28, 1.77), <0.001
Pathogens identified		
No	Reference group	Reference group
Yes	0.79 (0.71, 0.89), <0.001	0.63 (0.59, 0.68), <0.001
Principal site of infection		
Respiratory	1.17 (1.01, 1.36), 0.044	1.14 (1.04, 1.25), 0.007
Abdominal	Not applicable	Not applicable
Genitourinary	0.55 (0.45, 0.68), <0.001	0.53 (0.47, 0.60), <0.001
Soft tissue	0.70 (0.51, 0.97), 0.030	0.82 (0.68, 0.99), 0.047
Procedure-related	1.01 (0.85, 1.21), 0.895	0.72 (0.64, 0.81), <0.001
Others/Not specified	1.75 (1.47, 2.06), <0.001	1.67 (1.51, 1.84), <0.001
No. organ dysfunctions		
1	Reference group	Reference group
2	2.54 (2.19, 2.95), <0.001	2.17 (1.98, 2.37), <0.001
>2	5.51 (4.78, 6.36), <0.001	4.88 (4.48, 5.32), <0.001

OR, odds ratio; 95% CI, 95% confidence interval.

**Table 4:** Incidence and mortality trends of sepsis in adults aged 20–44 years.

Variable	2006	2015	Change Whole Period (%)	Annual Average Percent Change (%, 95% CI)
Rates				
Adjusted incidence ^‡^				
Overall	13.5	17.1	26.7	1.5 (0, 3.0)
Women	10.3	15.9	54.4	3.8 (2.1, 5.5) ^†^
Men	16.5	18.2	10.3	−0.2 (−1.6, 1.4)
Charlson Index (%)				
0	38.6	44.6	15.5	1.3 (0.6, 1.9) ^†^
>0	61.4	55.4	−9.8	−0.9 (−1.3, −0.4) ^†^
No. organ dysfunctions (%)				
1	48.0	45.0	−6.2	−0.6 (−1.4, 0.2)
2	28.9	23.9	−17.3	−1.6 (−2.4, −0.9) ^†^
>2	21.7	24.9	14.7	0.6 (−0.8, 2.1)
CRF (%)				
Overall	31.1	18.6	−40.2	−5.9 (−6.6, −5.2) ^†^
Sex				
Women	27.9	14.6	−47.7	−6.6 (−7.8, −5.4) ^†^
Men	33.0	21.9	−33.6	−5.1 (−6.1, −4.2) ^†^
Charlson’s index				
0	19.0	9.9	−47.9	−8.1 (−10.1, −6.1) ^†^
>0	38.7	25.6	−33.8	−4.7 (−5.2, −4.2) ^†^
No. organ dysfunctions				
1	18.6	11.0	−40.9	−6.6 (−8.7, −4.4) ^†^
2	33.9	17.7	−47.8	−7.0 (−8.2, −5.8) ^†^
>2	55.1	35.7	−35.2	−4.6 (−5.4, −3.9) ^†^

^†^ The Annual percent change is significantly different from zero (Poisson regression, *p* < 0.05). ^‡^ per 100,000 persons.
